# Supplementary figures and images for: Population-specific equations of age-related maximum handgrip force: a comprehensive review
Source: PeerJ. 2024 Jul 22;12:e17703. doi: 10.7717/peerj.17703 (PMC11271657; doi:10.7717/peerj.17703)

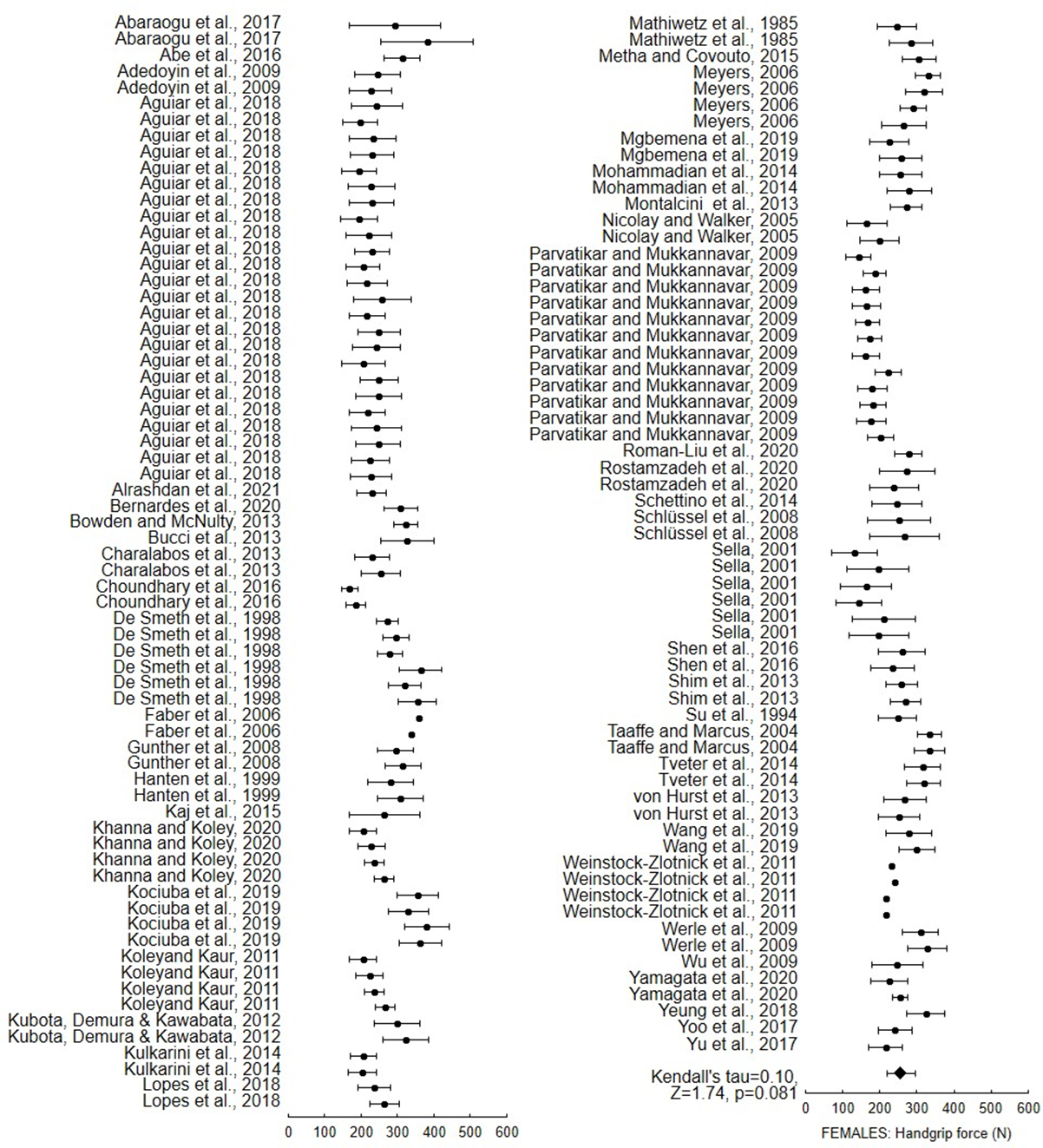

Supplement: Supplemental Information 2 [file peerj-12-17703-s002.png]

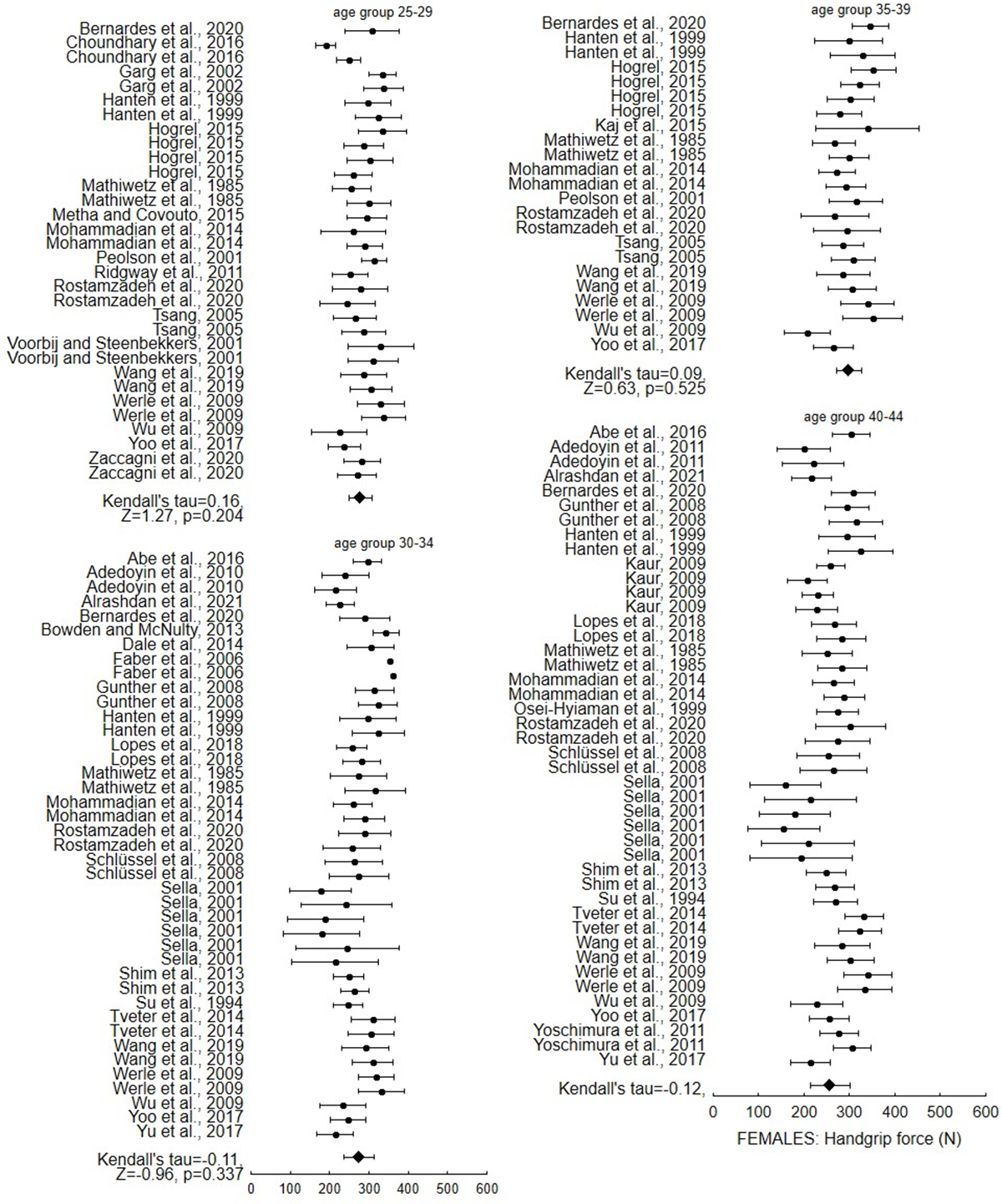

Supplement: Supplemental Information 3 [file peerj-12-17703-s003.png]

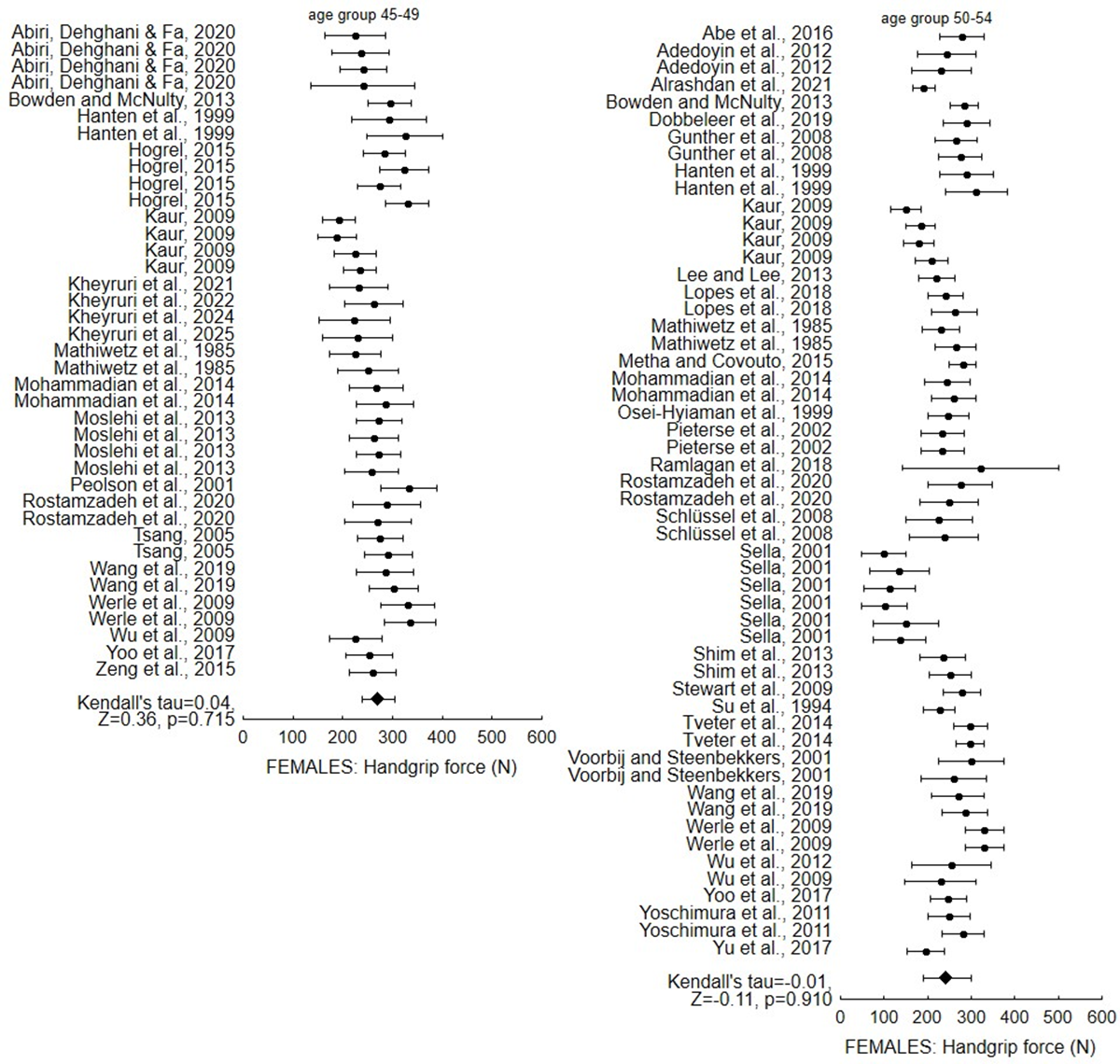

Supplement: Supplemental Information 4 [file peerj-12-17703-s004.png]

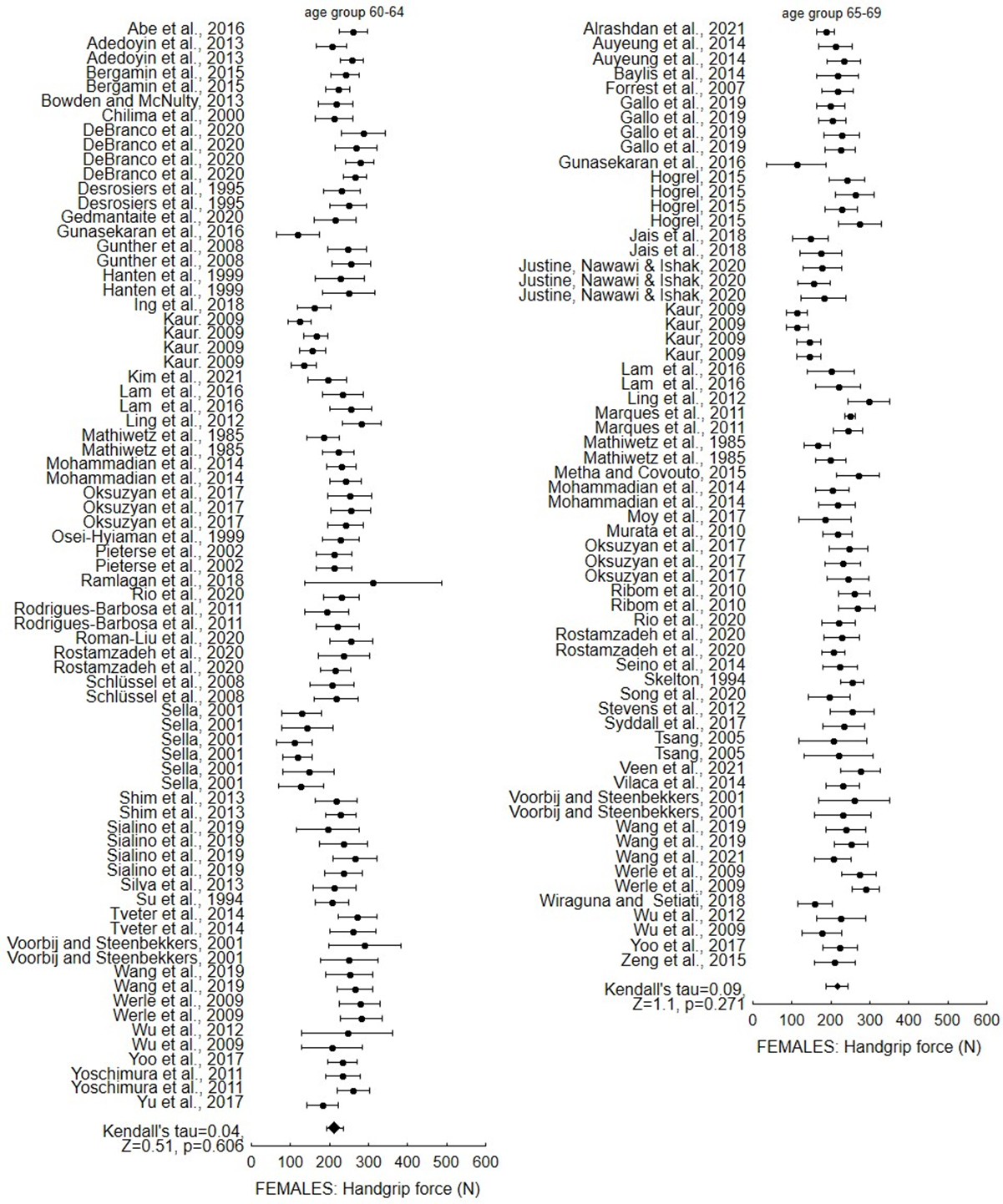

Supplement: Supplemental Information 5 [file peerj-12-17703-s005.png]

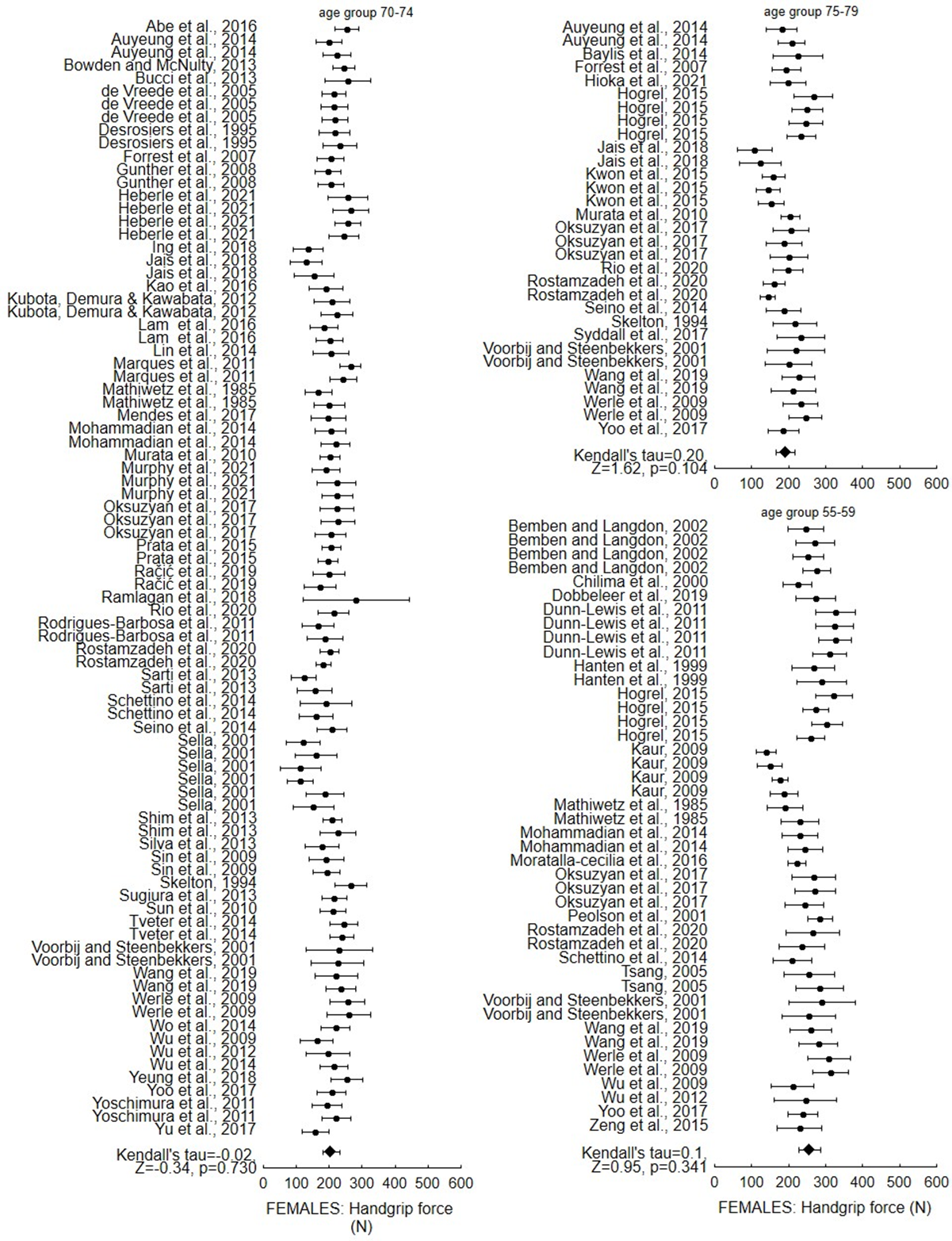

Supplement: Supplemental Information 6 [file peerj-12-17703-s006.png]

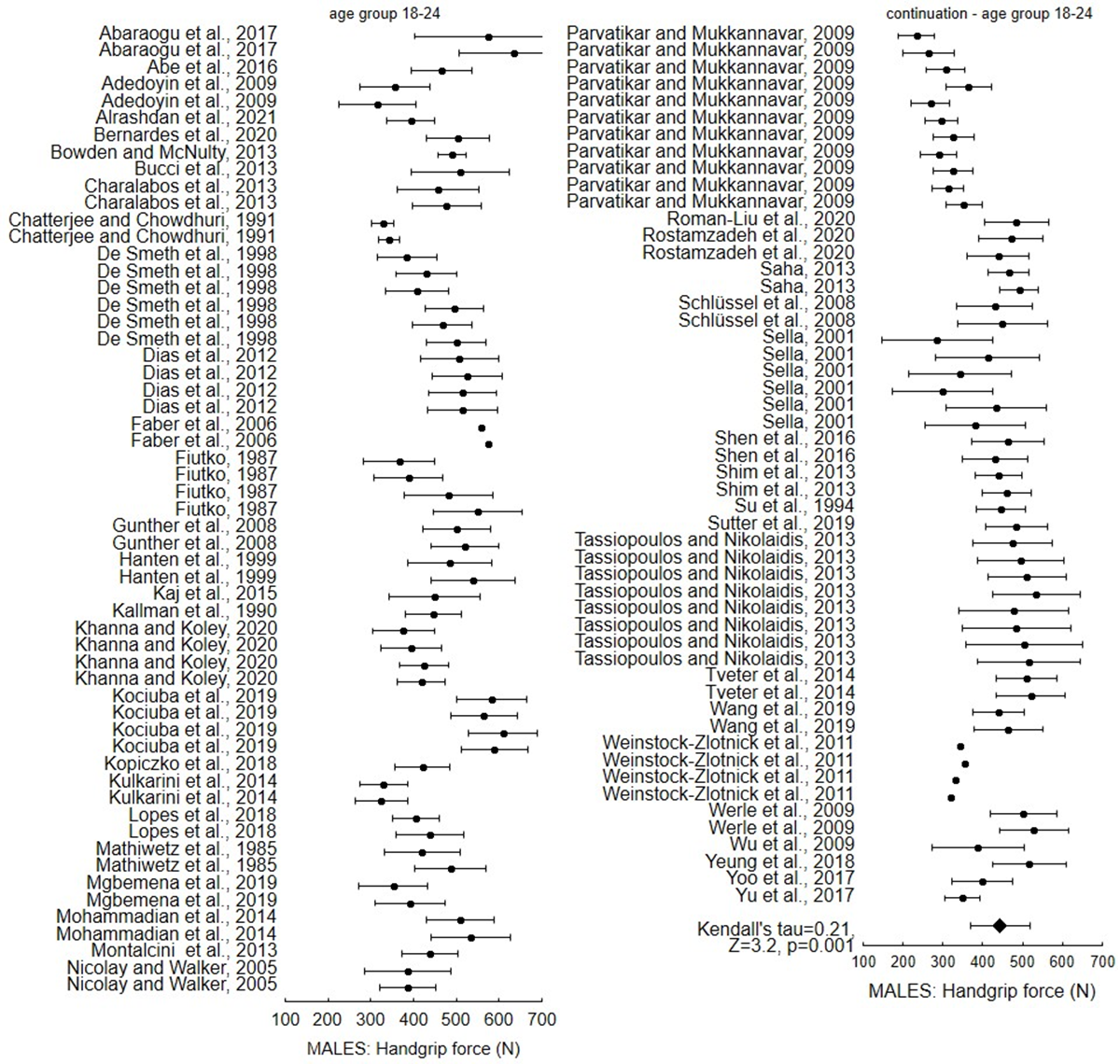

Supplement: Supplemental Information 7 [file peerj-12-17703-s007.png]

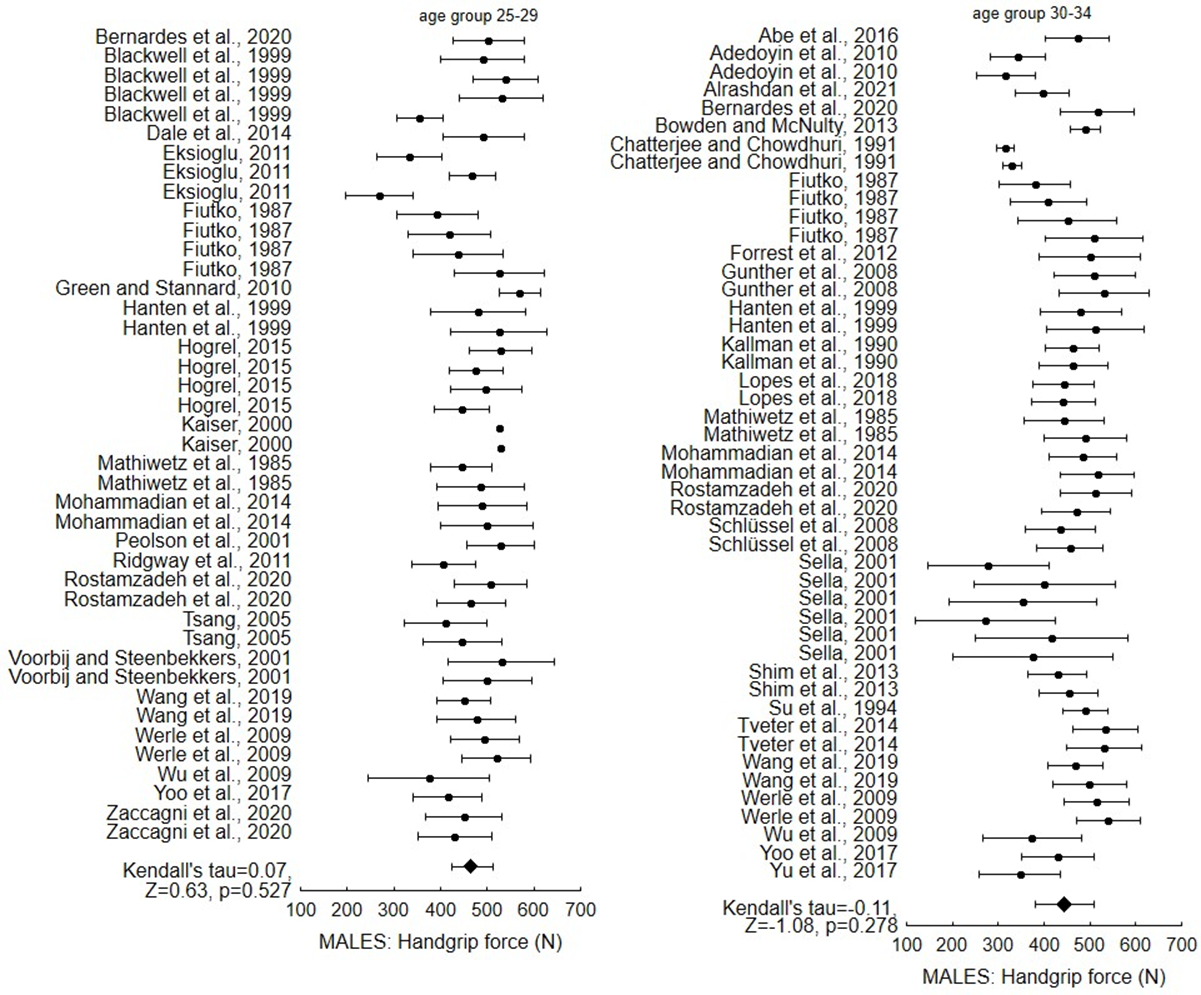

Supplement: Supplemental Information 8 [file peerj-12-17703-s008.png]

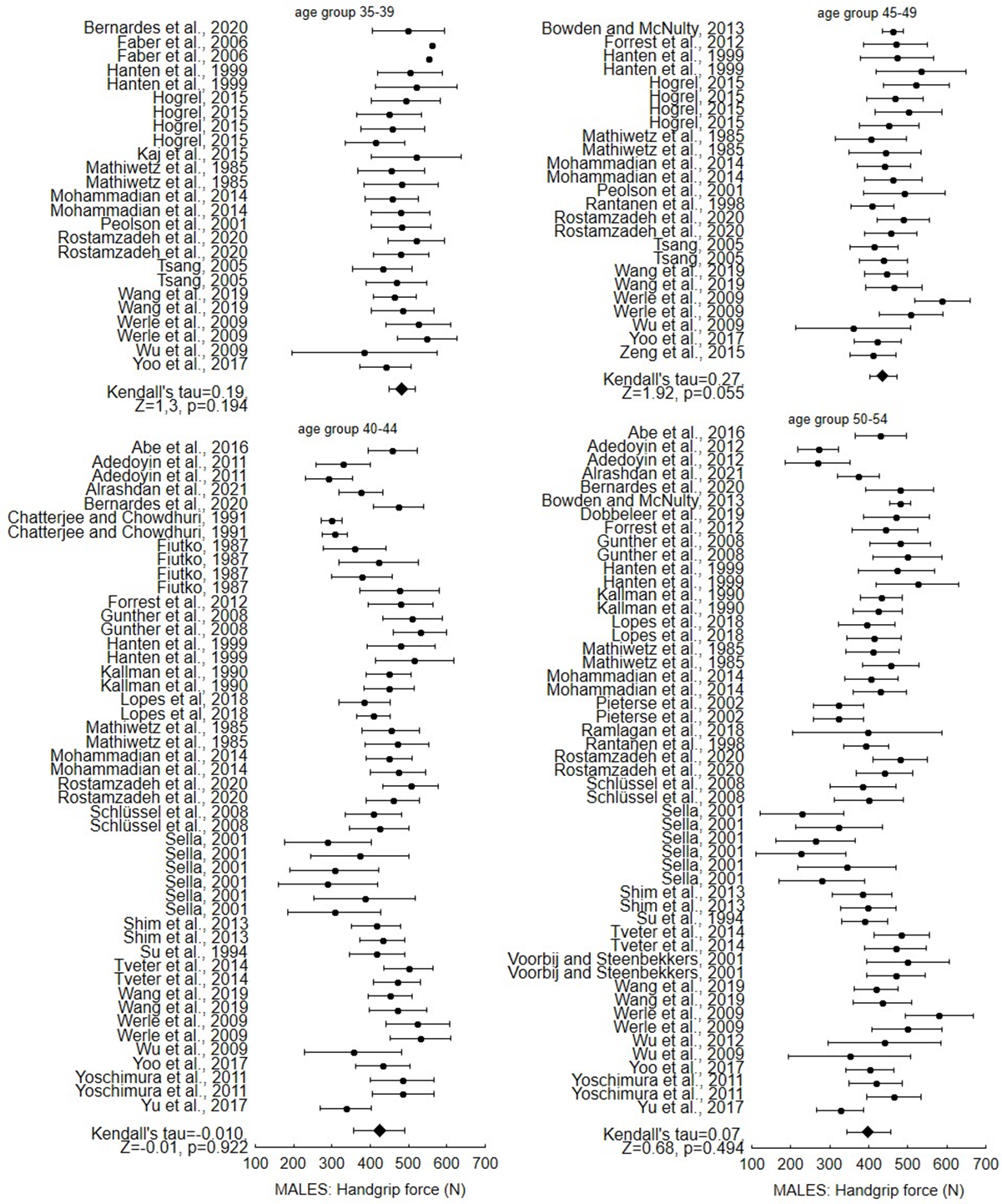

Supplement: Supplemental Information 9 [file peerj-12-17703-s009.png]

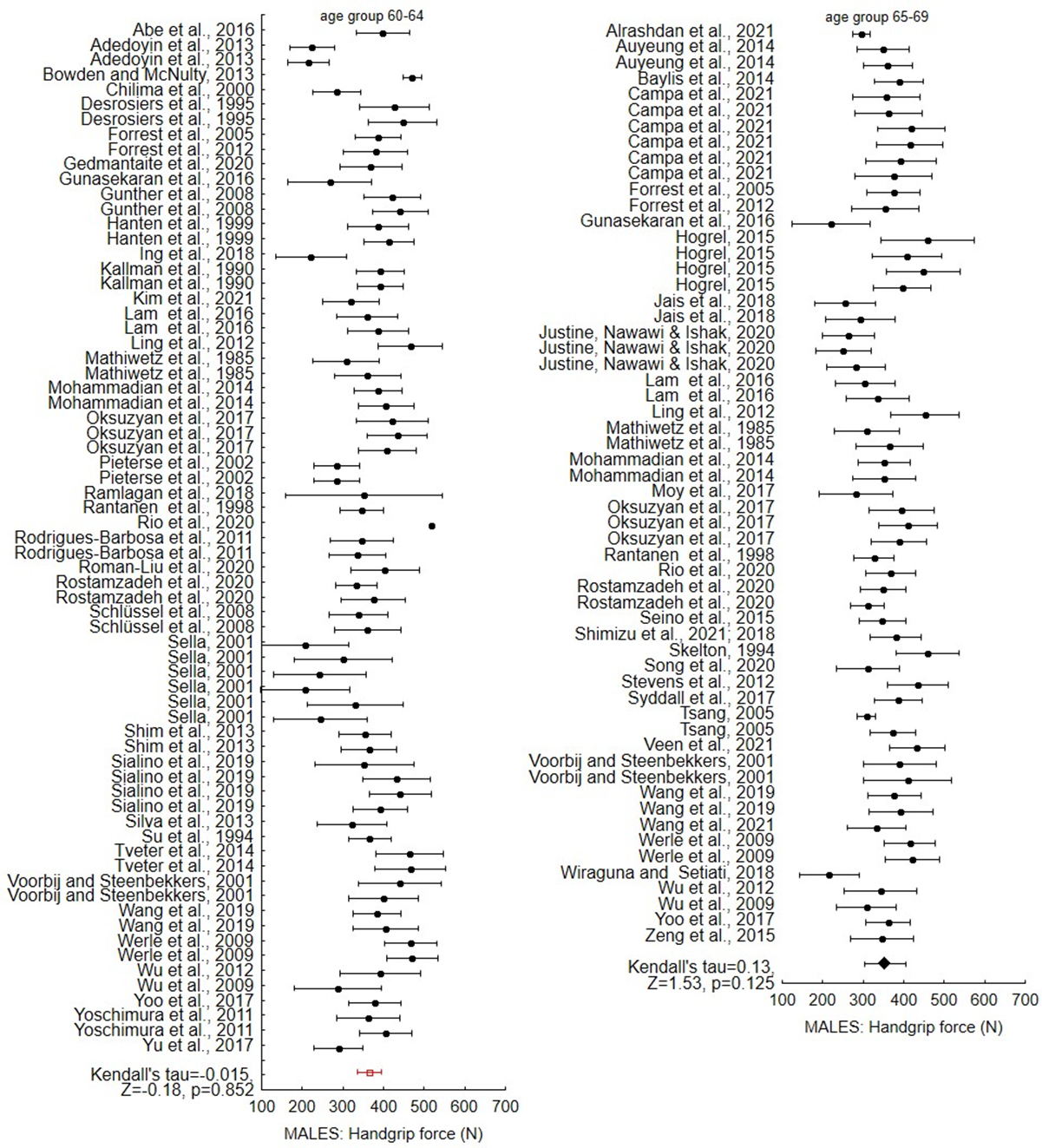

Supplement: Supplemental Information 10 [file peerj-12-17703-s010.png]

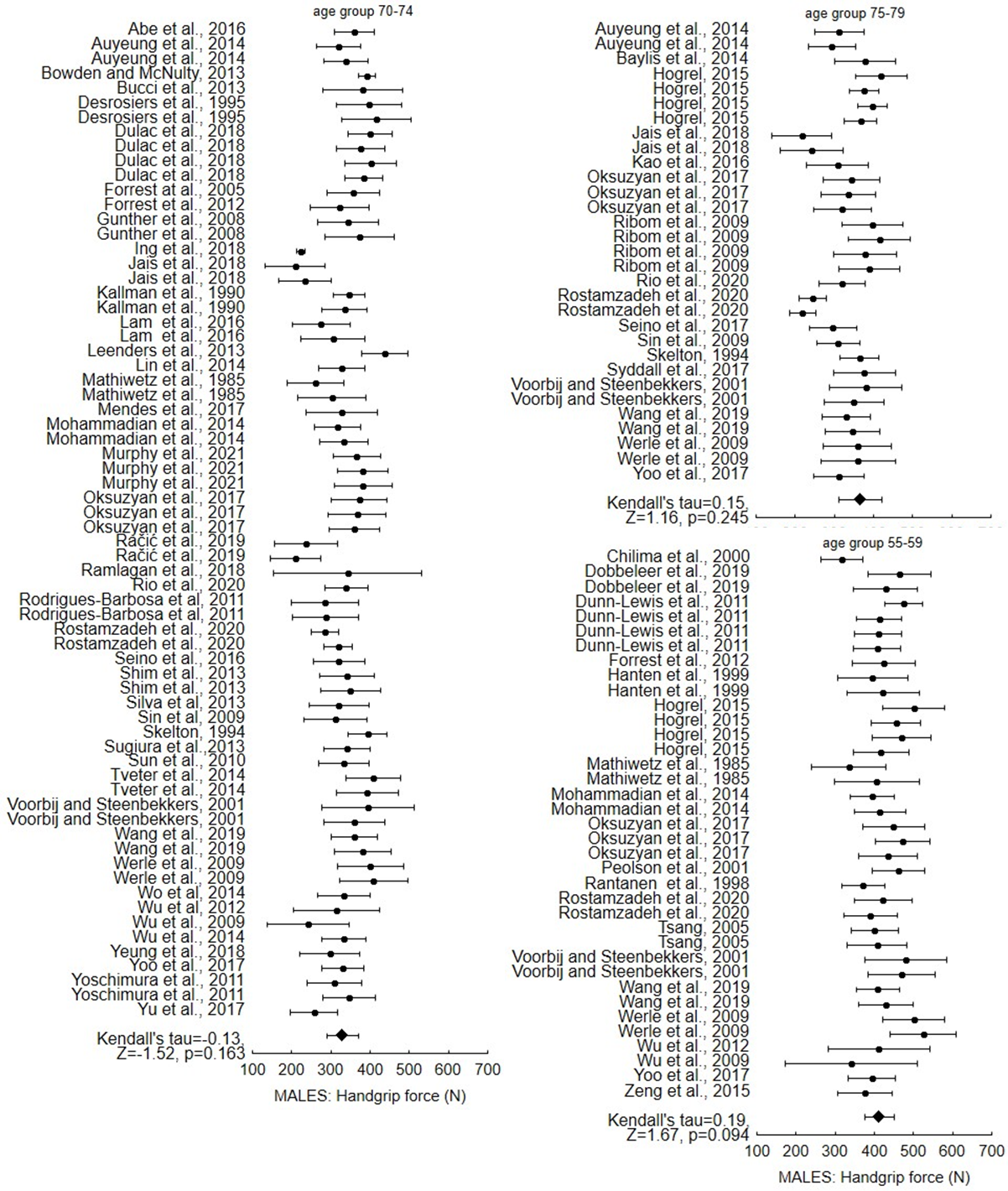

Supplement: Supplemental Information 11 [file peerj-12-17703-s011.png]
